# Supplementary material for: Impact of the 294 bp SINE Insertion in 5′UTR of the GLYATL3 Gene on Gene Expression and Phenotypic Variation
Source: Animals (Basel). 2025 May 9;15(10):1375. doi: 10.3390/ani15101375 (PMC12108459; doi:10.3390/ani15101375)
Supplement: Supplementary file 1 [file animals-15-01375-s001.zip › animals-3554457-supplementary.pdf]

Table S1. Information on animals and samples used.

Pig breeds	Type	Number	Tissue	Origin
**Duroc**	Commercial pig	24	Ear	Xuzhou,jiangsu
**Landrace**	Commercial pig	23	Ear	Xuzhou,Jiangsu
**Largewhite**	Commercial pig	458	Ear	Hefei,Anhui
**Sushan**	Hybrid pig	24	Ear	Taizhou,Jiangsu
**Sujiang**	Hybrid pig	24	Ear	Taizhou,Jiangsu
**Erhualian**	Native pig	24	Ear	Suzhou,Jiangsu
**Jiangquhai**	Native pig	23	Ear	Taizhou,Jiangsu
**Mi**	Native pig	24	Ear,Cerebellum,Pallium,Liver, Back fat	Changzhou,Jiangsu
**Meishan**	Native pig	24	Ear	Suzhou,Jiangsu
**Min**	Native pig	24	Ear	Heilongjiang,Dongbei
**Rongchang**	Native pig	24	Ear	Rongchang,Chongqing
**Tibetan**	Native pig	24	Ear	Kangding,Sichuan
**Jinhua**	Native pig	24	Ear	Hangzhou,Zhejiang
**Wuzhishan**	Native pig	23	Ear	Wuzhishan,Hainan
**Bama**	Native pig	24	Ear	Nanning,Guangxi

Table S2. Primers for SINE RIP.

Primers	Sequence	Tm/°C	Production/bp
GLYATL3-F<br>GLYATL3-R	AAATCAATCCGTTTTTGGTATG<br>TGTTGAATTTCATTTCTCCTCTT	58	965
GLYATL3-cds-F<br>GLYATL3-cds-R	CACCCTGGAAAAAGTGTTGAGG<br>CTCGCTCTGCAGTCCTTGTAT	60	295
SINE-MluI-F<br>SINE-EcoRI-R	acgcgtGTCAGTACCTCTGC<br>gaattcGTCCTCCAGTGGAAAGAGGA	62	306
Pro-KpnI-F<br>Pro-MluI-R	GCGGCCGCTTAAATACGTGACA<br>acgcgtCCCATATAGATTTTTATCTGTTCTGAGTAAA	62	1717
Pro+SINE-KpnI-F<br>Pro+SINE-MluI-R	GCGGCCGCTTAAATACGTGACA<br>TCCTCTTTCCACTGGAGGACgaattc	62	2011
